# Magnetron-Sputtered Polytetrafluoroethylene-Stabilized Silver Nanoisland Surface for Surface-Enhanced Fluorescence

**DOI:** 10.3390/nano10040773

**Published:** 2020-04-16

**Authors:** Martin Šubr, Petr Praus, Anna Kuzminova, Eva Kočišová, Ondřej Kylián, Franck Sureau, Marek Procházka, Josef Štěpánek

**Affiliations:** 1 Institute of Physics, Faculty of Mathematics and Physics, Charles University, Ke Karlovu 5, 121 16 Prague, Czech Republicpraus@karlov.mff.cuni.cz (P.P.); kocisova@karlov.mff.cuni.cz (E.K.);; 2 Department of Macromolecular Physics, Faculty of Mathematics and Physics, Charles University, V Holešovičkách 2, 180 00 Prague, Czech Republic; annakuzminova84@gmail.com (A.K.); Ondrej.Kylian@mff.cuni.cz (O.K.); 3 Laboratoire Jean Perrin, Sorbonne University, Case Courrier 114, 4 Place Jussieu, 75005 Paris, France; franck.sureau@upmc.fr

**Keywords:** surface-enhanced fluorescence (SEF), time-resolved, riboflavin, lifetime, enhancement factor

## Abstract

Surface-enhanced fluorescence (SEF) requires the absorption/emission band of the fluorophore, the localized surface plasmon resonance (LSPR) of the nanostructure and the excitation wavelength to fall in the same (or very close) spectral range. In this paper, we monitor the SEF intensity and lifetime dependence of riboflavin (vitamin B2) adsorbed on a spacer-modified Ag substrate with respect to the thickness of the spacer. The substrates were formed by silver nanoislands deposited onto magnetron-sputtered polytetrafluoroethylene (ms-PTFE). The spacer was formed by the ms-PTFE layer with the thickness ranging from ~5 to 25 nm. The riboflavin dissolved in dimethylsulfoxide (DMSO) at a 10 µM concentration forms, at the ms-PTFE surface, a homogeneous layer of adsorbed molecules corresponding to a monomolecular layer. The microspectroscopic measurements of the adsorbed layer were performed through a sessile droplet; our study has shown the advantages and limitations of this approach. Time-resolved fluorescence enabled us to determine the enhanced fluorescence quantum yield due to the shortening of the radiative decay in the vicinity of the plasmonic surface. For the 5 nm ms-PTFE layer possessing the largest (estimated 4×) fluorescence enhancement, the quantum yield was increased 2.3×.

## 1. Introduction

Fluorescence detection represents a well-established technique used in a wide range of biochemical and biomedical applications. To date, various detection schemes (both intrinsic and extrinsic) based on fluorescence such as direct detection, sandwich, competitive or inhibition assay were proposed. The sensitivity of fluorescence to the local environment such as pH, gas concentration or a presence of a specific ligand has led to the development of many sophisticated sensing formats, making use of the effect of fluorescence quenching, changes in fluorescence anisotropy or the resonant transfer of energy [1]. Unlike Raman or infrared absorption spectroscopy, fluorescence is a two-step process, and thus fluorescence sensing is not only limited to stationary analysis but also time-resolved measurements which provide valuable information about the lifetime of the probes. Although the fluorescence cross-section is generally higher in comparison to the other optical processes mentioned above, the need for reduced detection limits is still opposed by the low quantum yield of some fluorophores, their photobleaching or problems with the background emissions from the sample. These limitations may be partly overcome by surface-enhanced fluorescence (SEF), which represents one of the three main surface-enhanced spectroscopic methods besides surface-enhanced Raman scattering (SERS) and surface-enhanced infrared absorption (SEIRA) [2,3,4].

The current understanding of SEF attributes the enhancement effect to the resonance excitation of surface plasmons at the metal–dielectric interface [5,6,7,8,9,10]. When the fluorophore is placed in the vicinity of the nanostructured metal surface, it is subjected to a much stronger electromagnetic field in comparison with the same molecule in free space. Due to the more concentrated electromagnetic field around the nanoparticle, the excitation rate of the fluorophore is higher and also the emission rate of the fluorophore is usually increased. These two mechanisms are difficult to separate and the proportion between them remains unclear, but it is expected to depend on the spectral overlap between the absorption/emission band of the studied molecule, the excitation wavelength and their mutual position within the localized surface plasmon resonance (LSPR) band of a given nanostructure [11,12,13,14]. However, the enhancement effect for fluorophores placed on metal nanostructures is competitive with the radiationless quenching caused by an energy transfer at the metal–dielectric interface, which is more pronounced at very short distances between the fluorophore and the metal surface (up to ~2–3 nm) [3,4,5,8,15]. Thus, there exists an optimum distance for which the enhancement effect is most significant (usually in the range between 5 and 20 nm) and a spacer must be used to keep the interspace between the metal and the fluorophore [16]. However, because of the distance-dependent electromagnetic effect and the role of fluorescence quenching, the maximum fluorescence enhancement factor, typically 10^0^–10^3^ [3,5,7,8], can hardly match the typical values of the Raman enhancement factor [15].

Detection of SEF requires the absorption/emission band of the fluorophore, the LSPR peak of the nanostructures and the excitation wavelength to fall in the same (or very close) spectral range. Due to this, silver, gold and copper nanostructures are the most suitable metals for SEF as they exhibit LSPR in the visible spectral range. In addition, it is well known that the plasmon resonance properties of such metallic nanostructures can be controlled by optimizing the nanoparticle dimensions, their geometry and composition [13,17,18,19,20]. Different spacing was used in the literature to keep the optimum distance, such as silicon oxides [6,13,16,21], polymers [22,23], proteins [11,24] or DNA strands [12,25,26]. However, the correct interpretation of the enhancement mechanism and the competing enhancing/quenching processes requires a thorough investigation of dependence on the spacer thickness as well as the plasmonic properties, and a correlation between the SEF enhancement and the lifetime shortening is usually needed. Although all these approaches have appeared in the literature already in the 1980s [6,7], most of these aspects so far have been studied separately. 

In this contribution, we systematically monitor the SEF intensity and lifetime dependence of riboflavin (vitamin B2) adsorbed on a spacer-modified nanostructured Ag substrate with respect to the thickness of the spacer. The enhanced fluorescence of riboflavin on a highly reduced graphene oxide (rGO) surface was reported previously [27]. In our study, the enhancing surface was formed by Ag nanoislands deposited onto magnetron-sputtered polytetrafluoroethylene (ms-PTFE). These substrates hold great promise for analytical applications due to their SERS performance, excellent reproducibility, time stability, ease of preparation and cost effectiveness [28]. Moreover, this deposition procedure may be easily adapted for the fabrication of surfaces with LSPR conditions gradually changing along the sample length [17]. Their plasmonic properties, which may cover the whole visible spectral range, can be used for the identification of the best overlap of the absorption/emission band of the fluorophore with the LSPR maximum of the nanostructures. For the spacer, we use an ms-PTFE layer with the thickness ranging from ~5 to 25 nm. 

In previously published SEF studies, the fluorophore was deposited at the SEF-active surface via evaporation of a drop of the fluorophore solution or via rinsing the wafer with the SEF-active surface in the fluorophore solution and subsequent raising [6,7,8,9,10,11,12,13,14,15,16]. Both procedures resulted in large inhomogeneity of the fluorophore distribution on the surface. Moreover, it is difficult to determine the effect of the surface if we compare the fluorescence on the surface in contact with the air and the fluorescence of the molecules in the solution. In our study, we measured fluorescence from the surface directly through the sitting droplet, i.e., under the conditions of a natural dynamic equilibrium between the adsorbed molecules and the molecules in the solution within the droplet. An important advantage of this approach is also the heat transfer to the droplet reservoir, which significantly reduces the photodegradation rate of an analyte in comparison with measurements on a dried surface. However, this approach has brought about some new questions to be answered. They concern the relative proportion of the violating fluorescence signal from the droplet volume or the worsening of vertical resolution due to the passage of the beams through the droplet.

## 2. Materials and Methods 

### 2.1. Studied Biomolecule

**Figure 1:**
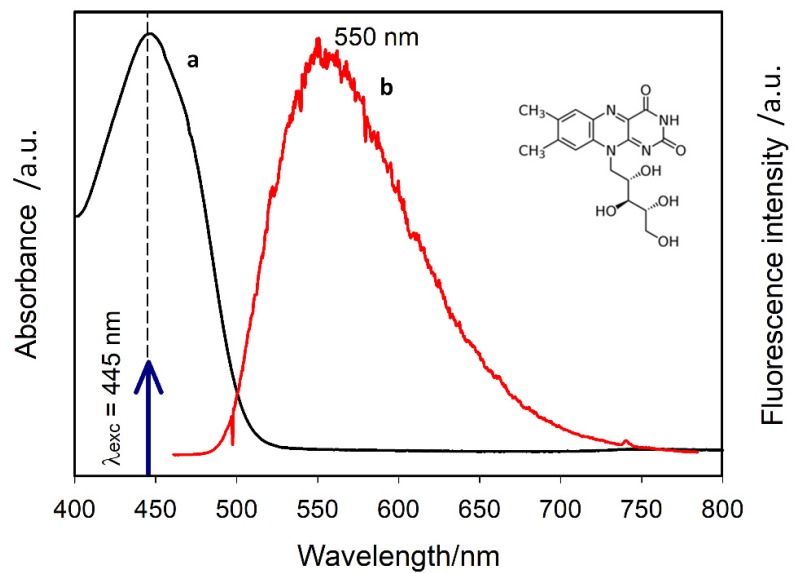
Absorption (**a**) and fluorescence (**b**) spectrum of riboflavin solution in water. The vertical arrow indicates the excitation wavelength. The chemical formula of riboflavin is shown as an inset.

Riboflavin (vitamin B2, Sigma-Aldrich, Prague, Czech Republic) was used as a model fluorophore biomolecule. It absorbs light in the spectral region below ~500 nm (Figure 1), i.e., in the spectral region corresponding to the LSPR of our structures (see below). Two different solvents, dimethylsulfoxide (DMSO) and water, were used to prepare the riboflavin solutions of the final riboflavin concentration 10^−5^ M (pH ~6 for the aqueous solution). 

### 2.2. Fabrication of the SEF Nanostructures

**Figure 2:**
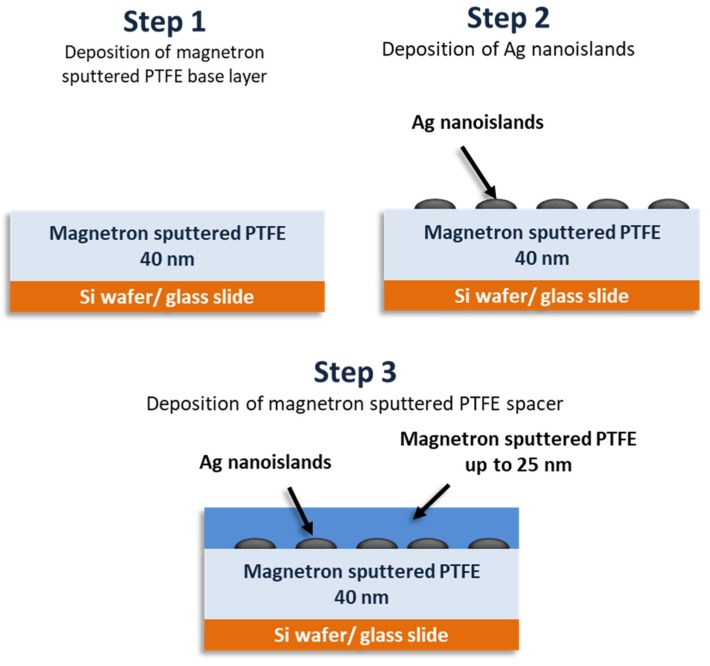
Three-step deposition of surface-enhanced fluorescence (SEF) nanostructures.

**Figure 3:**
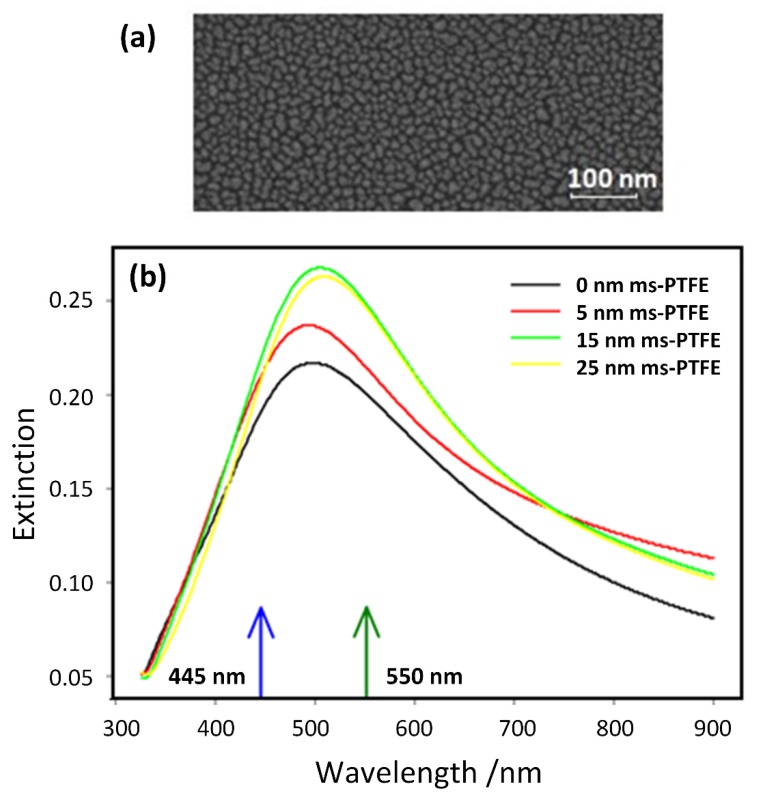
(**a**) Scanning electron microscopy (SEM) image of a typical Ag nanoisland layer, (**b**) extinction spectra of the Ag nanoislands overcoated with magnetron-sputtered polytetrafluoroethylene (ms-PTFE) layers of various thicknesses. The arrows indicate the excitation wavelength (blue) and the wavelength of the fluorescence maximum (green).

The SEF nanostructures were prepared on polished Si wafers (~3 × 1 cm) as well as on glass slides for the measurements of their extinction spectra. The fabrication procedure (see Figure 2) was carried out based on a step-by-step optimization process described in our previous work [28]. Low-pressure magnetron sputtering of the Ag target in an argon atmosphere was employed for the deposition of the Ag nanoislands (responsible for the enhancing effect). The Ag nanoislands are, in this case, formed during the growth of a thin Ag film below the percolation threshold. As the formation and resulting morphology of growing the Ag nanoislands is highly sensitive to the substrate material, the Ag nanoislands were deposited over the ms-PTFE film (thickness 40 nm) to assure the same surface for both the Si and glass substrates. The ms-PTFE was also used as a spacer to keep the riboflavin molecules away from making close contact with the nanoislands. The thickness of the ms-PTFE overcoat ranged between 5 and 25 nm, as measured by the spectroscopic ellipsometry (Woollam M-2000DI, Woollam M-2000DI, Lincoln, NE, USA). The deposition time of the Ag nanoislands was set at 22 s, corresponding to a broad extinction peak centered around 500 nm, overlapping both the absorption and the emission band of the fluorophore. The typical scanning electron microscopy (SEM) image (TESCAN Mira 3.15 kV accelerating voltage, Brno, Czech Republic) of the formed Ag nanoislands is presented in Figure 3a. According to the statistical analysis of acquired SEM images, the mean equivalent disk radius of the Ag nanoislands was around 5 nm (but this is close to the resolution limit of the employed SEM). After the deposition of the ms-PTFE cover layer, the maximum peak of the plasmon resonance slightly moved to the higher wavelengths and the intensity of the peaks slightly increased. This is demonstrated in Figure 3b, where the extinction spectra (UV–Vis spectrophotometer Hitachi U-2910, Tokyo, Japan) are shown for the samples with ms-PTFE layers of various thicknesses analogous to those used in the measurements of the riboflavin SEF but prepared on glass slides. 

### 2.3. Fluorescence Measurements

A confocal microspectrofluorimeter adapted for time-resolved fluorescence measurements by using a phase-modulation principle with homodyne data acquisition was employed to obtain the SEF spectra and to determine the fluorescence lifetimes [29,30,31,32]. Its simplified block diagram is shown in Figure S1. The fluorescence lifetime was determined simultaneously for all emission wavelengths by acquiring several even phase-shifted spectra of the modulated excitation laser beam in relation to the fixed modulation phase shift of the detector gain. The fluorescence lifetime was calculated from the frequency-dependent phase shift and the intensity demodulation [29,30,31,32] for the modulation frequencies evenly covering a 5–200 MHz interval. A laser diode module (Omicron LDM 442.50.A350, Rodgau-Dudenhofen, Germany) with a sinusoidal intensity modulation (50 mW peak output, attenuated to one to tens of µW at the sample) was used for the excitation at a 445 nm wavelength. A confocal epifluorescence upright microscope (Zeiss UMSP–80, Jena, Germany) with a 10× objective (X10 Zeiss Ultrafluar with a numerical aperture of 0.2, Jena, Germany) was used to excite the fluorescence (see inset of Figure S1). The collected SEF signal was focused on the entrance slit of the Jobin–Yvon HR640 spectrograph (Paris, France) equipped with a 100 line/mm grating. The spectral detection window (375 nm-wide) covered both the excitation wavelength (detected elastic scattering was used as a lifetime reference) and the emission spectrum of the fluorophore. A more detailed description of the microspectrofluorimeter setup and the phase-resolved spectra processing can be found in [32]. Measurements of the fluorescence enhancement factor and spectral reproducibility were carried out by using a confocal Raman upper microscope (Witec, Alpha 300, Ulm, Germany) with XYZ piezoelectric positioners by mapping inside the droplet on 100 points over a ~100 × 100 µm surface area. An average intensity was determined for each Z position of the microscope confocal point.

## 3. Results and Discussion

### 3.1. SEF Measurements

In contrast to a confocal microscopic measurement underneath a plane-parallel layer, which is known to be affected by the degradation of both the lateral and vertical resolution, this worsening is much smaller in a droplet. The curvature of the droplet surface reduces the incident angles significantly, and thus the aberrations of the refracted rays. In an ideal case of a hemisphere droplet shape, the resolution should not alter at all if the focus is placed on the hemisphere center. 

**Figure 4:**
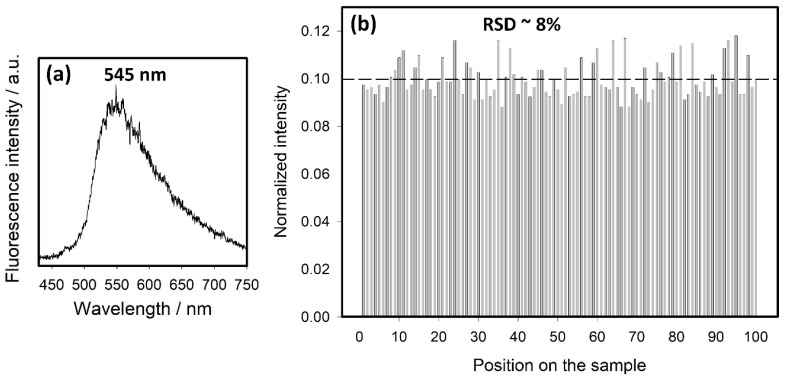
(**a**) Typical SEF spectrum of riboflavin, (**b**) reproducibility of the SEF measurements, determined by spectral mapping inside the droplet on 100 points over a ~100 × 100 µm surface area. The average intensity is indicated by the dashed line. The thickness of the ms-PTFE overlay of the Ag nanoislands was 5 nm in both experiments.

The droplets in our experiments were of a slightly different shape, but we realized only the marginal effects of a lower resolution. We did not notice any difficulty in focusing on the SEF-active surface. We aimed to center the objective lens laterally above the top of the drop (via the incident light reflection), nevertheless, we found out the insensitivity of the measured fluorescence intensity to small lateral shifts. The typical rough SEF spectrum of riboflavin is shown in Figure 4a. It is broadened and slightly blue-shifted in comparison to the fluorescence spectrum from the solution (Figure 1, spectrum b). We suppose that the broadening is due to the distribution of the molecules in the droplet (from bulk and surface), and that the blue shift is due to the interaction of the molecules within the Ag surface. Spectral mapping within a 100 × 100 µm area possessed fluorescence intensities with about 8% relative standard deviation (RSD) (Figure 4b). This result simultaneously confirms the high homogeneity of the layer of the riboflavin molecules adsorbed from the droplet. 

The size of the droplets we used was limited by a requirement of having no significant evaporation of the droplet during the experiment. For the time-resolved fluorescence measurements lasting 10 to 20 min, we realized that the smallest usable volume of the droplets was 5 µL for the DMSO solution and 10 µL for the aqueous solution, respectively.

### 3.2. SEF Intensities and Lifetimes

**Figure 5:**
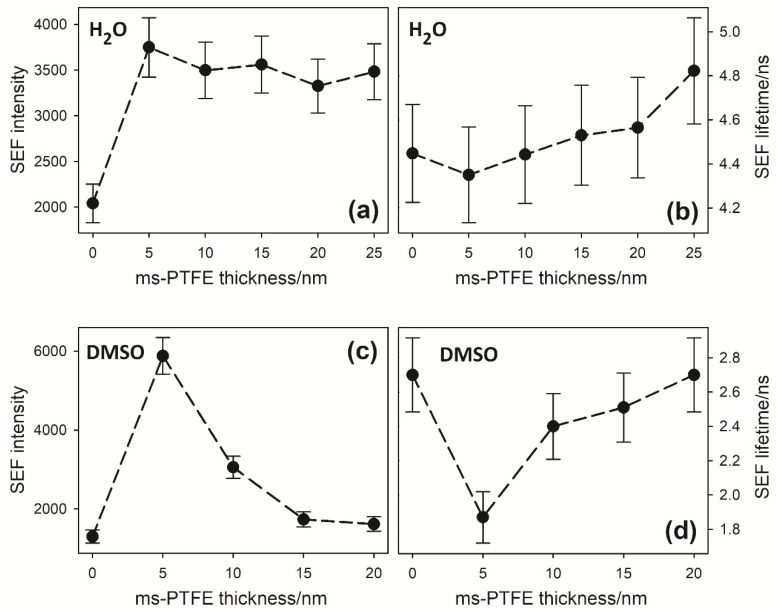
Fluorescence intensity (**a**,**c**) and lifetime (**b**,**d**) of riboflavin adsorbed on the SEF-active surface from a sessile droplet obtained for various thicknesses of the ms-PTFE overlay of the Ag nanoislands. Solvents: water (**a**,**b**) and dimethylsulfoxide (DMSO) (**c**,**d**). The error bars indicate the estimated precision as a standard deviation. The lines connecting the adjacent points are only a support for the eye and do not reflect the dependence of the measured quantities on the thickness of the overlay.

The time-resolved fluorescence measurements were carried out for the SEF surfaces with ms-PTFE overlays of various thicknesses. The fluorescence intensity profiles and corresponding lifetimes obtained from the averaged 4–5 independent measurements are shown in Figure 5. The fluorescence intensity was determined as the height of the riboflavin emission band above the spectral background. The precision of the lifetime values was estimated considering the variations in the results obtained for the particular modulation frequencies. That of the fluorescence intensity was derived taking into account the precision of the background level, the effect of the signal noise and the effect of the inhomogeneity over the surface demonstrated in Figure 4.

In both solvents, DMSO and water, the dependencies of the fluorescence intensity on the ms-PTFE overlay thickness follow the same characteristic pattern, with a maximum at the layer thickness of 5 nm. By further increasing the overlay thickness, the effect of the distance from the nanostructured Ag layer is noticeable, resulting in a gradual decrease. The lowest fluorescence intensity is observed for the surface without the ms-PTFE overlay. 

The dependence of the fluorescence lifetime on the overlay thickness corresponds to that of the fluorescence intensity, i.e., the lifetime is minimal for the 5 nm overlay and increases with the further increase in the thickness. It clearly demonstrates that the fluorescence intensity is enhanced, at least partly, by the increase in the fluorescence quantum yield due to a higher radiative decay rate in the vicinity of the plasmonic Ag layer. It is assumed [4,5,9] that the fluorescence lifetime, *τ*_F_, which is in a solution given by the inverse of the sum of radiative (*Γ*) and nonradiative (*k*_nr_) decay rates as

(1) $$\tau_{F}=\frac{1}{\Gamma +k_{\mathrm{nr}}}$$

is shortened in the close vicinity of a metal surface due to the increase in both the radiative decay rate (from *Γ* to *Γ* + *Γ*_m_) and the nonradiative decay rate (from *k*_nr_ to *k*_nr_ + *k*_m_). Index m means “metal”. The fluorescence lifetime for a molecule close to the metal is then

(2) $$\tau_{F,m}=\frac{1}{\Gamma +\Gamma_{m}+k_{\mathrm{nr}}+k_{m}}$$

The vicinity of a metal surface influences in the same manner as the fluorescence quantum yield, which is given by a ratio of the radiative and the total decay rates, while for a molecule in a solution the quantum yield is

(3) $$Q_{0}=\frac{\Gamma }{\Gamma +k_{\mathrm{nr}}}$$

and the proximity of metal changes the quantum yield to

(4) $$Q=\frac{{\Gamma +\Gamma }_{m}}{{\Gamma +\Gamma }_{m}+k_{\mathrm{nr}}+k_{m}}$$

Taking into account that the nanostructured Ag layer can enhance the local electromagnetic excitation field, leading to an increased absorption of the fluorophore, the total fluorescence enhancement in respect to the measurement in an environment without the effect of the proximity of plasmonic metal layer is [3]

(5) $$\xi =\frac{I_{SEF}}{I_{0}}=EF\cdot \frac{Q}{Q_{0}}=EF\cdot \frac{{\Gamma +\Gamma }_{m}}{{\Gamma +\Gamma }_{m}+k_{\mathrm{nr}}+k_{m}}\cdot \frac{\Gamma +k_{\mathrm{nr}}}{\Gamma }$$

where *EF* is an increase in the absorption intensity.

*Γ* and *k*_nr_ are intrinsic fluorophore characteristics in a given environment and can be determined by measuring the molecule in an appropriate solvent. The fluorescence lifetime of riboflavin in an aqueous solution we measured was *τ*_F_ = (5.0 ± 0.1) ns, which is in agreement with the previously published data in [33,34]. The value of the radiative decay rate reported therein is *Γ* = 5.6 × 10^7^ s^−1^, which gives *k*_nr_ = 14 × 10^7^ s^−1^ and *Q*_0_ = 0.27, according to Equations (1) and (3). For the DMSO solution, the analogously obtained values are *τ*_F_ = (2.9 ± 0.1) ns, *Γ* = 7.4 × 10^7^ s^−1^, *k*_nr_ = 2.7 × 10^8^ s^−1^ and *Q*_0_ = 0.22.

The parameters *EF, Γ*_m_, and *k*_nr_ represent the surface effects that depend, in a non-trivial way, on the fluorophore distance from the plasmonic metal surface. Since these phenomena have contradictory effects on the fluorescence intensity, the total distance-dependent profile results from a competition between the amplification of the local field and the increase in the quantum yield with the enhanced radiationless energy transfer at very short distances. It is assumed that *k*_m_ is negligible for distances >~4–5 nm [35].

The changes of the fluorescence intensity and lifetime in Figure 5 demonstrate the phenomenon of SEF of the adsorbed riboflavin molecules for the distances of 5 nm or more from the nanostructured Ag layer. In these cases, the effect of *k_m_* can be neglected and the variables occurring in Equations (1)–(5) can be unambiguously computed (Table 1). In this instance, the ratio *Q*/*Q*_0_ is higher than 1, which means that fluorescence is enhanced. However, because of the limited range of the electromagnetic effect, the enhancement decreases rapidly with a further increasing *d*.

On the other hand, the rate of nonradiative decay is greatly enhanced when the fluorophore and the metal surface are closely contacted, which results in fluorescence suppression. However, the values obtained for the surface without any spacer (ms-PTFE thickness = 0) do not seem to be fully consistent with the relationships for the fluorescence of molecules on the metal surface. For DMSO, the obtained lifetime value corresponds to that of a molecule in a solution and the intensity is close to the limit value for a high thickness of the layer when the surface effect should be very weak. In the case of water, the fluorescence intensity for the surface without ms-PTFE cover is lower than the high thickness limit, but still represents about 60% of this value. This indicates that the obtained fluorescence characteristics are not attributable exclusively to the molecules adsorbed at the surface but are the result of signal summation from the adsorbed molecules and those dissolved in the drop volume. For the quantitative analysis, it is thus necessary not only to estimate the ratios of the adsorbed and dissolved molecules but also the ratio of the fluorescence contribution from the surface and the bulk. We demonstrated that this is possible based on certain additional experiments and considerations. They are described below in brief; for details see the Supplementary Information.

### 3.3. The Droplet Geometry

The droplet possesses the shape of a spherical cap, the geometrical parameters of which are determined by its volume and the contact angle. Table 2 summarizes the measured contact angles of the droplets used in our experiments and the calculated ratio ζ of the contact area with the surface to the droplet volume. The contact angles for a particular solute and various thicknesses of the ms-PTFE layer did not vary within an experimental error.

### 3.4. Estimation of the Amount of the Riboflavin Molecules Adsorbed on the Surface

Fifty droplets of the 10 µM riboflavin solution in DMSO, each of 5 µL volume, were placed on the ms-PTFE surface (5 nm thickness). After several minutes, a sufficient time for achieving an equilibrium between the adsorbed and dissolved molecules, the content of the droplets was gently suctioned and the remaining riboflavin concentration was determined by an absorption measurement. It was found that the adsorption caused about a 50% decrease in the riboflavin concentration inside the droplets. An analogous experiment with 10 µL droplets of the riboflavin aqueous solution did not exhibit any detectable concentration decrease. Considering the possible effects of water evaporation from the droplets during the experiment, it can be concluded that the adsorption did not reduce the riboflavin concentration inside the droplets by more than 15% in the aqueous solution.

Knowing the ratio ζ between the surface area in contact with the droplet and the droplet volume, we can calculate the surface density of the adsorbed molecules. For DMSO it was 2.0 × 10^12^ molecules per mm^2^. Taking into account the dimensions of the riboflavin molecule, this value seems to correspond to a monomolecular layer. For water, we only conclude that the surface density was less than 1.4 × 10^12^ molecules per mm^2^, i.e., it did not exceed the value of a monomolecular layer.

These conclusions were supported by another experiment, when we measured the intensity of the fluorescence signal for droplets of different volumes on the substrate with a 5 nm ms-PTFE overlay. For the DMSO solution, we obtained the satisfactory agreement of the experimental results with a theoretical curve based on an assumption of a Langmuir adsorption isotherm, valid for the formation of the first monolayer [36]. In the case of the aqueous solution, the results indicated that the ratio between the signal from the surface and the signal from the volume was remarkably lower in comparison with DMSO.

### 3.5. Estimation of the Ratio of Fluorescence Signals from the Surface Area and the Volume of the Droplet

**Figure 6:**
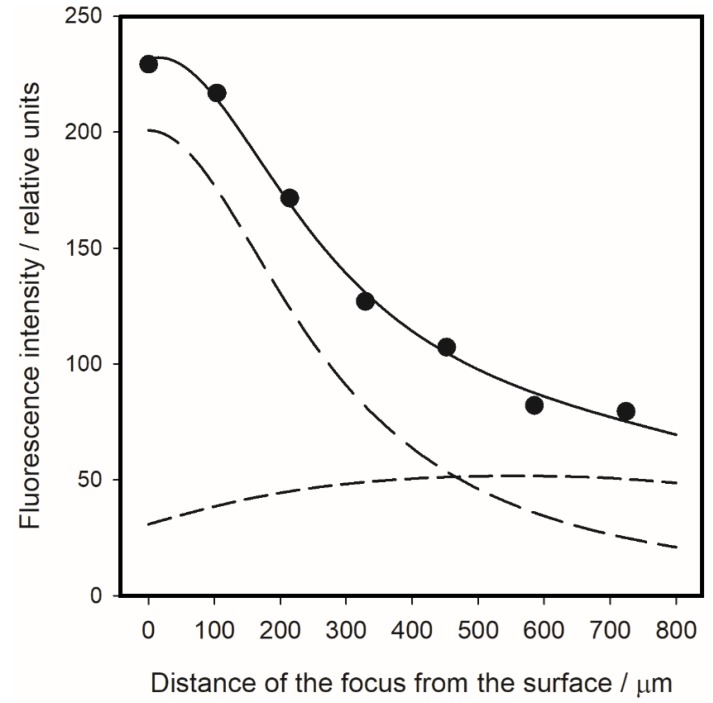
Dependence of the fluorescence intensity at 585 nm on the increasing focus distance from the surface. 5 µL droplet of riboflavin solution in DMSO on the substrate with a 5 nm ms-PTFE overlay (dots). The focus position was recalculated from the position of the objective considering refraction at the spherical droplet surface. Lines show results of the least-square fit: the total fluorescence signal (solid), the signal from the surface (longer dash) and the signal from the droplet volume (shorter dash).

We have performed an experiment in which the distance of the microscope objective was gradually increased, starting with a focus on the surface. Figure 6 shows the obtained dependence of the fluorescence intensity (at the emission band maximum) on the focus distance from the surface for the 5 µL of riboflavin solution in DMSO on the substrate with a 5 nm ms-PTFE overlay.

The substantial decrease in the fluorescence signal when the focus distance to the surface increases demonstrates clearly that the signal from the molecules adsorbed on the surface is dominant. By the simple consideration that, at a sufficiently long distance from the surface, the signal from the surface should completely disappear while the signal from the volume should be doubled, we can conclude without a complicated analysis that the signal contribution from the volume does not, for focus at the surface, exceed 17%.

More exact results can be obtained by a least-square fit employing the theoretical model (see Supplementary Information for details) based on an assumption (generally accepted) that the fluorescence intensity from the elementary horizontal layers of the solution depends on their vertical distance from the focus as

(6) $$\frac{dI_{vol}\left(z\right)}{dz}=\frac{\varphi_{0}}{1+{\left(\frac{z}{\delta }\right)}^{2}}$$

Figure 6 shows that the fit based on this dependence matches the experimental data very well. The fit provided the halfwidth of the Lorentzian function (Equation (6)) to be *δ* = 0.27 mm, and the ratio of the surface and volume signals for focus at the surface to be 6.5:1. This means that the volume signal represents only 13% of the total fluorescence signal in the case of the 5 nm thick ms-PTFE overlay. Moreover, as the riboflavin concentration inside the drop and its surface density is known, the model allows us to estimate the ratio of fluorescence signals from the surface and the solution per one molecule. The value of this total enhancement factor is for the 5 nm ms-PTFE overlay *ξ* = *I*_SEF_/*I*_0_ = 4.0. This gives the factor of the increase in absorption intensity to be (according to Equation (5) and Table 1) EF = 1.7.

An analogous experiment, when the fluorescence intensity was measured for various focus distances from the surface, was performed for a 10 µL drop of riboflavin aqueous solution. The obtained dependence was, however, quite different. The fluorescence intensity gradually increased with the increasing distance to a limit of approximately double the starting value (data not shown). This clearly demonstrates that, in this case, the amount of adsorbed riboflavin molecules is indeed very low and that the majority of the fluorescence measured through the sessile droplet comes from the bulk in the case of the aqueous solution. This, of course, significantly affected the results of the time-resolved fluorescence measurements and caused the fluorescence intensity and lifetime (Table 1) to differ only marginally for the various thicknesses of the ms-PTFE cover. The most remarkable effect is the decrease in the fluorescence intensity for the uncovered Ag island surface. It can be explained by a dynamical quenching of the dissolved riboflavin molecules in the vicinity of the surface or by the substantially more effective adsorption on an uncovered surface, which decreased the concentration inside the droplet while the fluorescence of the adsorbed molecules was completely quenched.

## 4. Conclusions

It has been revealed that riboflavin dissolved in DMSO at a 10 µM concentration forms, on the ms-PTFE surface, a homogeneous layer of adsorbed molecules, the surface density of which corresponds to a monomolecular layer. Time-resolved fluorescence measurements carried out through a sessile droplet for the Ag island surfaces covered by ms-PTFE films of various thicknesses have shown the SEF for thicknesses of 5 nm and higher, while for the bare Ag nanoislands, the generally expected nonradiative quenching was confirmed. The lifetime measurements enabled us to determine the enhanced fluorescence quantum yield due to the shortening of the radiative decay in the vicinity of the plasmonic surface. This effect was the main factor of the fluorescence enhancement but the enhanced absorption probability due to the electromagnetic field enhancement was also found to be significant. For the 5 nm ms-PTFE layer possessing the largest (estimated 4×) fluorescence enhancement, the quantum yield was increased 2.3×.

Our results have also shown the possibility of microspectroscopic measurements of adsorbed layers through a sessile droplet and demonstrated the advantages and limitations of this approach. The measurements were performed at the homogeneous layer of the adsorbed molecules corresponding to an equilibrium between the adsorbed and dissolved molecules. Ancillary measurements of the signal dependence on the distance of the microscope focus from the surface can determine the proportion of the signal from the surface and of that from the droplet bulk and can even enable one to estimate the ratio of the signals relative to the molecular amounts. On the other hand, this approach does not provide quantitatively reliable data in the case of weak adsorption like in our case of an ms-PTFE surface and riboflavin aqueous solution.

## Figures and Tables

**Table 1:** Lifetime characteristics of riboflavin in respective environments and their implications on quantum yield enhancement (*Q/Q*_0_). Lifetimes were experimentally measured; all other characteristics were computed using Equations (1)–(5), neglecting the effect of *k*_m_.

Water					DMSO				
ms-PTFE Thickness	Lifetime	*τ_F,m_/τ_F_*	*Γ_m_*	*Q/Q*_0_	ms-PTFE Thickness	Lifetime	*τ_F,m_/τ_F_*	*Γ_m_*	*Q/Q*_0_
0 nm	4.45 ns	0.89	N/A	N/A	0 nm	2.70 ns	0.93	N/A	N/A
5 nm	4.35 ns	0.87	2.99	1.34	5 nm	1.87 ns	0.64	18.99	2.30
10 nm	4.44 ns	0.89	2.51	1.29	10 nm	2.40 ns	0.83	4.17	1.39
15 nm	4.53 ns	0.91	2.08	1.24	15 nm	2.51 ns	0.87	3.11	1.30
20 nm	4.57 ns	0.91	1.91	1.23	22 nm	2.70 ns	0.93	1.48	1.15
25 nm	4.82 ns	0.96	0.74	1.09					

**Table 2:** Geometric parameters of the measured droplets.

Surface	Solvent	Volume/µL	Contact Angle/°	ζ/mm^−1^
ms-PTFE layer	DMSO	5	71 ± 4	1.5 ± 0.1
ms-PTFE layer	water	10	104 ± 2	0.68 ± 0.03

## Supplementary Materials

The following are available online at https://www.mdpi.com/2079-4991/10/4/773/s1, Figure S1: Simplified block diagram of the fluorescence microspectrofluorimeter, Figure S2: The sessile droplet geometry, Figure S3: Dependence of the fluorescence signal on the droplet geometry, Figure S4: Focus position inside the droplet, Figure S5: Geometry of the experiment.

## References

[1] Lakowicz J.R. (2006). Principles of Fluorescence Spectroscopy.

[2] Procházka M. (2016). Surface-Enhanced Raman Spectroscopy: Bioanalytical, Biomolecular and Medical Applications.

[3] Aroca R.F. (2013). Plasmon enhanced spectroscopy. Phys. Chem. Chem. Phys..

[4] Le Ru E.C., Etchegoin P.G. (2009). Principles of Surface-Enhanced Raman Spectroscopy and Related Plasmonic Effects.

[5] Li J.F., Li C.Y., Aroca R.F. (2017). Plasmon-enhanced fluorescence spectroscopy. Chem. Soc. Rev..

[6] Wokaun A., Lutz H.P., King A.P., Wild U.P., Ernst R.R. (1983). Energy-Transfer in Surface Enhanced Luminescence. J. Chem. Phys..

[7] Weitz D.A., Garoff S. (1983). The Enhancement of Raman-Scattering, Resonance Raman-Scattering, and Fluorescence from Molecules Adsorbed on a Rough Silver Surface. J. Chem. Phys..

[8] Lakowicz J.R. (2001). Radiative decay engineering: Biophysical and biomedical applications. Anal. Biochem..

[9] Lakowicz J.R., Geddes C.D., Gryczynski I., Malicka J., Gryczynski Z., Aslan K., Lukomska J., Matveeva E., Zhang J., Badugu R. (2004). Advances in surface-enhanced fluorescence. J. Fluoresc..

[10] Fort E., Gresillon S. (2008). Surface enhanced fluorescence. J. Phys. D Appl. Phys..

[11] Tam F., Goodrich G.P., Johnson B.R., Halas N.J. (2007). Plasmonic enhancement of molecular fluorescence. Nano. Lett..

[12] Chen Y., Munechika K., Ginger D.S. (2007). Dependence of fluorescence intensity on the spectral overlap between fluorophores and plasmon resonant single silver nanoparticles. Nano. Lett..

[13] Stranik O., Nooney R., McDonagh C., MacCraith B.D. (2007). Optimization of nanoparticle size for plasmonic enhancement of fluorescence. Plasmonics.

[14] Bharadwaj P., Novotny L. (2007). Spectral dependence of single molecule fluorescence enhancement. Opt. Express.

[15] Sun G., Khurgin J.B. (2012). Origin of giant difference between fluorescence, resonance, and nonresonance Raman scattering enhancement by surface plasmons. Phys. Rev. A.

[16] Mishra H., Buddha L.M., Karolin J., Dragan A.I., Geddes C.D. (2013). Experimental and theoretical study of the distance dependence of metal-enhanced fluorescence, phosphorescence and delayed fluorescence in a single system. Phys. Chem. Chem. Phys..

[17] Hanuš J., Libenská H., Khalakhan I., Kuzminova A., Kylián O., Biederman H. (2017). Localized surface plasmon resonance tuning via nanostructured gradient Ag surfaces. Mater. Lett..

[18] Kelly K.L., Coronado E., Zhao L.L., Schatz G.C. (2003). The optical properties of metal nanoparticles: The influence of size, shape, and dielectric environment. J. Phys. Chem. B.

[19] Liaw J.W., Tsai H.Y., Huang C.H. (2012). Size-Dependent Surface Enhanced Fluorescence of Gold Nanorod: Enhancement or Quenching. Plasmonics.

[20] Vahl A., Strobel J., Reichstein W., Polonskyi O., Strunskus T., Kienle L., Faupel F. (2017). Single target sputter deposition of alloy nanoparticles with adjustable composition via a gas aggregation cluster source. Nanotechnology.

[21] Asselin J., Legros P., Gregoire A., Boudreau D. (2016). Correlating Metal-Enhanced Fluorescence and Structural Properties in Ag@SiO2 Core-Shell Nanoparticles. Plasmonics.

[22] Ferreira M., Constantino C.J., Olivati C.A., Vega M.L., Balogh D.T., Aroca R.F., Faria R.M., Oliveira O.N. (2003). Langmuir and Langmuir-Blodgett films of poly[2-methoxy-5-(n-hexyloxy)-p-phenylenevinylene]. Langmuir.

[23] Constantino C.J.L., Aroca R.F., Mendonça C.R., Mello S.V., Balogh D.T., Oliveira O.N. (2001). Surface enhanced fluorescence and Raman imaging of Langmuir-Blodgett azopolymer films. Spectroc. Acta A Molec. Biomolec. Spectr..

[24] Bardhan R., Grady N.K., Cole J.R., Joshi A., Halas N.J. (2009). Fluorescence Enhancement by Au Nanostructures: Nanoshells and Nanorods. ACS Nano.

[25] Dragan A.I., Bishop E.S., Casas-Finet J.R., Strouse R.J., McGivney J., Schenerman M.A., Geddes C.D. (2012). Distance Dependence of Metal-Enhanced Fluorescence. Plasmonics.

[26] Zhang J., Fu Y., Chowdhury H., Lakowicz J.R. (2007). Metal-enhanced single-molecule fluorescence on silver particle monomer and dimer: Coupling effect between metal particles. Nano Lett..

[27] Iliut M., Gabudean A.M., Leordean C., Simon T., Teodorescu C.M., Astilean S. (2013). Riboflavin enhanced fluorescence of highly reduced graphene oxide. Chem. Phys. Lett..

[28] Šubr M., Petr M., Kylián O., Kratochvíl J., Procházka M. (2015). Large-scale Ag nanoislands stabilized by a magnetron-sputtered polytetrafluoroethylene film as substrates for highly sensitive and reproducible surface-enhanced Raman scattering (SERS). J. Mat. Chem. C.

[29] Praus P., Sureau F. (2000). Spectral decomposition of intracellular complex fluorescent signals using multiwavelength phase modulation lifetime determination. J. Fluoresc..

[30] Praus P., Kocisova E., Seksek O., Sureau F., Stepanek J., Turpin P.Y. (2007). Advanced microfluorescence methods in monitoring intracellular uptake of "antisense" oligonucleotides. Curr. Org. Chem..

[31] Kočišová E., Praus P., Rosenberg I., Seksek O., Sureau F., Štĕpánek J., Turpin P.Y. (2004). Intracellular uptake of modified oligonucleotide studied by two fluorescence techniques. Biopolymers.

[32] Kočišová E., Praus P., Bok J., Bonneau S., Sureau F. (2015). Intracellular Monitoring of AS1411 Aptamer by Time-Resolved Microspectrofluorimetry and Fluorescence Imaging. J. Fluoresc..

[33] Drossler P., Holzer W., Penzkofer A., Hegemann P. (2003). Fluorescence quenching of riboflavin in aqueous solution by methionin and cystein. Chem. Phys..

[34] Zirak P., Penzkofer A., Mathes T., Hegemann P. (2009). Photo-dynamics of roseoflavin and riboflavin in aqueous and organic solvents. Chem. Phys..

[35] Waldeck D.H., Alivisatos A.P., Harris C.B. (1985). Nonradiative Damping of Molecular Electronic Excited-States by Metal-Surfaces. Surf. Sci..

[36] Masel R. (1996). Principles of Adsorption and Reaction on Solid Surfaces.

