# Supplementary material for: Magnetron-Sputtered Polytetrafluoroethylene-Stabilized Silver Nanoisland Surface for Surface-Enhanced Fluorescence
Source: Nanomaterials (Basel). 2020 Apr 16;10(4):773. doi: 10.3390/nano10040773 (PMC7221687; doi:10.3390/nano10040773)
Supplement: Supplementary file 1 [file nanomaterials-10-00773-s001.pdf]

Supplementary Information

Magnetron-sputtered Polytetrafluoroethylene-stabilized Silver Nanoisland Surface for Surface-Enhanced Fluorescence

Martin Šubr[1], Petr Praus[1], Anna Kuzminova[2], Eva Kočišová[1], Ondřej Kylián[2], Franck Sureau[3], Marek Procházka[1,*] and Josef Štěpánek[1]

[1]Institute of Physics, Faculty of Mathematics and Physics, Charles University, Ke Karlovu 5, 121 16 Prague, Czech Republic
[2]Department of Macromolecular Physics, Faculty of Mathematics and Physics, Charles University, V Holešovičkách 2, 180 00 Prague, Czech Republic
[3]Laboratoire Jean Perrin, Sorbonne University, Case Courrier 114, 4 Place Jussieu, 75005 Paris, France

Fluorescence measurement:

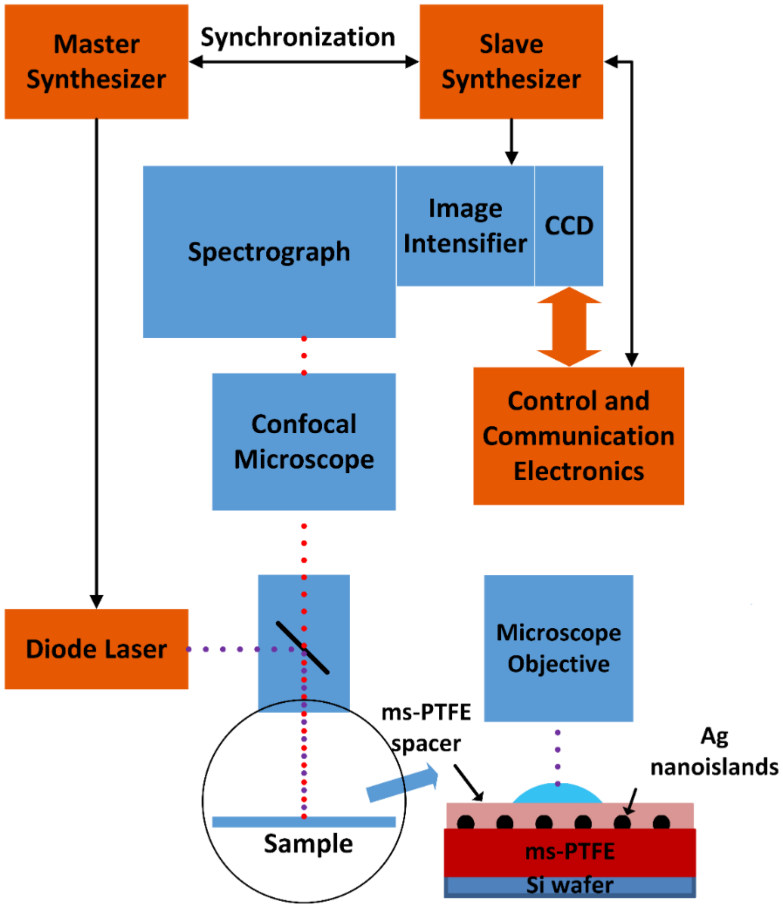

Figure S1. Simplified block diagram of the fluorescence microspectrofluorimeter with a 2D spectral and time resolution. Inset: Scheme of the surface-enhanced fluorescence (SEF) signal collection.

Sessile droplet geometry

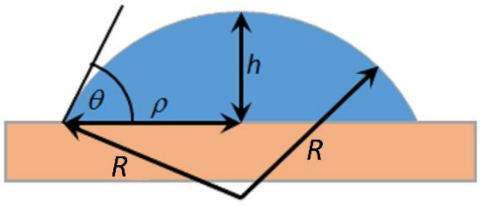

Figure S2. The sessile droplet geometry.

Up to a certain volume limit that was not exceeded, the sessile droplet takes the shape of a spherical cap. Its geometrical parameters, i.e., the radius (*R*) of the sphere, the contact base radius (*ρ*) and the height of the cap (*h*) are given by the droplet volume (*V*) and the contact angle (*θ*):

$$R = \left[\frac{3\,V}{\pi\,(1-\cos\theta)^2(2+\cos\theta)}\right]^{1/3} \qquad \text{(S1)}$$

$$,\ \rho = R\ \sin\theta \qquad ,\ h = R\,(1-\cos\theta) \quad . \qquad \text{(S1)}$$

The ratio of the contact circle area to the droplet volume is

$$\zeta = \frac{\pi\rho^2}{V} = \left[\frac{9\,\pi\,(\sin\theta)^2}{V\,(1-\cos\theta)^4(2+\cos\theta)^2}\right]^{1/3} \qquad \text{(S2)}$$

Surface density determined from the concentration decrease inside the droplet

The concentration decrease (Δc) inside the droplet caused by the adsorption leads to an average surface density of the adsorbed molecules (N_A is the Avogadro's number):

$$\sigma = \frac{\Delta c\,V\,N_A}{\pi\rho^2} = \frac{\Delta c\,N_A}{\zeta} \qquad \text{(S3)}$$

Dependence of the fluorescence signal on the droplet volume

The intensity of the fluorescence signal was measured for droplets of different volumes on the substrate with a 5 nm overlay. To minimize the effect of the sessile droplet evaporation, we used the confocal microspectrometer Witec (the disadvantage, however, was that we could not take the obtained ratio of the volume to the surface signal into the analysis of the time-resolved measurement). Figure S3 shows the results for the dimethylsulfoxide (DMSO) solution. The data were fitted with a theoretical curve based on an assumption of a Langmuir adsorption isotherm, valid for the formation of the first monolayer [36].

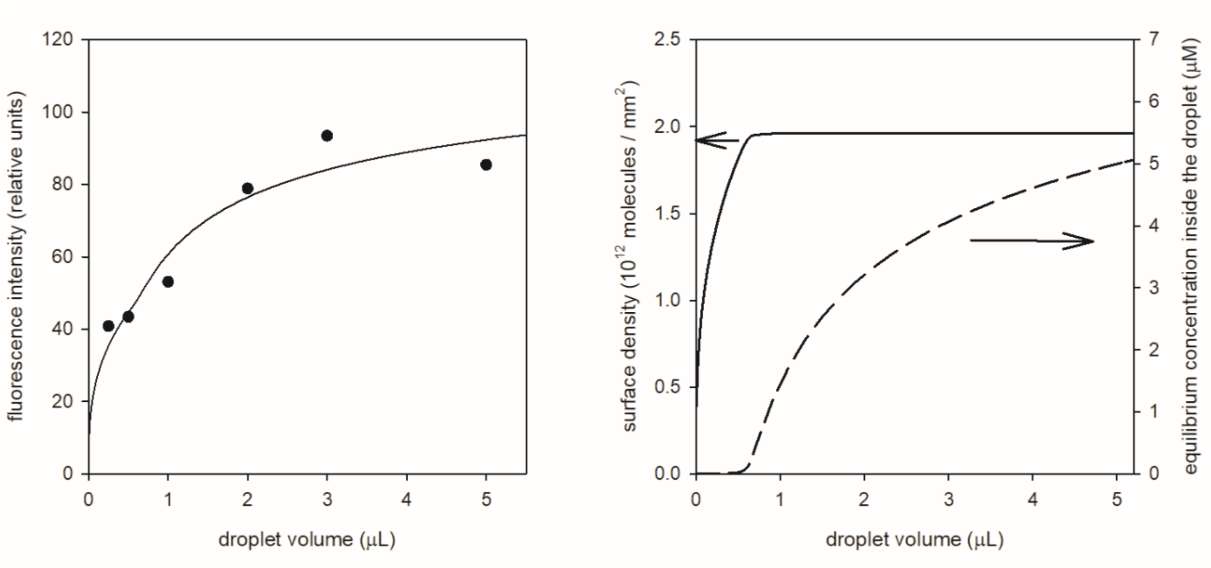

Figure S3. Left: The dependence of the fluorescence signal on the droplet volume. Fluorescence signal measured by the microspectrometer Witec from droplets of riboflavin solution in DMSO on the surface with a 5 nm ms-PTFE overlay (circles). The line shows the fit according to the formula for the Langmuir adsorption isotherm. Right: The riboflavin surface density (solid line) and its residual concentration in the droplet (dashed line) resulting from the fit.

The surface density depends then on the residual concentration of the adsorbate inside the droplet (c′) as

$$\sigma = \sigma_0 \frac{\beta\, c'}{1 + \beta\, c'} \tag{S4}$$

where σ_0 is the surface density of the first molecular monolayer. For a given coefficient β, the σ_0 value was calculated so that σ was 2.0×10^{12} molecules/mm^2 for the 5 μL droplet (c' = 5 μM). The surface density and the corresponding concentration in the droplet volume are related as

$$c' + \frac{\sigma\,\zeta}{N_A} = c_0 \tag{S5}$$

where c_0 is the initial concentration of the solution. Relations S4 and S5 provide quadratic equations for σ and c' as functions of the Langmuir coefficient β and the droplet volume (through the ratio ζ of the droplet volume and the surface contact area):

$$\sigma = \frac{\beta\left(c_0 + \sigma_0 \frac{\zeta}{N_A}\right) + 1 - \sqrt{D}}{2\beta \frac{\zeta}{N_A}}\ , c' = \frac{\beta\left(c_0 - \sigma_0 \frac{\zeta}{N_A}\right) - 1 + \sqrt{D}}{2\beta} \tag{S6}$$

$$D = \left(c_0 - \sigma_0 \frac{\zeta}{N_A}\right)^2 + 2\beta\left(c_0 + \sigma_0 \frac{\zeta}{N_A}\right) + 1$$

The experimental data were least-square fitted by the theoretical dependence

$$I(V) = I_{ads}(V) + I_{vol}(V) = A\,\frac{\sigma(V)}{N_A} + B\,c'(V) \tag{7}$$

The free parameters of the fit were the value of the coefficient β and the two coefficients of proportionality between the fluorescence signal and the riboflavin surface density or the riboflavin concentration inside the droplet. The best fit was reached for the high values of β. It can be seen from

Figure S6 that in this case the surface density of a monomolecular is virtually reached for droplet volumes higher than 1 μL.

An analogous experiment for the riboflavin aqueous solution gave somewhat less quality data. Furthermore, the results of the fit did not satisfactorily match the experimental data, showing only that the signal from the volume should be substantially stronger than that from the surface.

Focus position inside the droplet

The focus position inside the droplet was calculated by a numerical integration of the beams across the objective area, assuming the Gaussian intensity profile (half of the maximum at the edge), followed by a determination of the intensity maximum along the vertical axis. The calculations were performed for the 5 μL DMSO (71° contact angle) and 10 μL water (104° contact angle) droplets (see Figure S4).

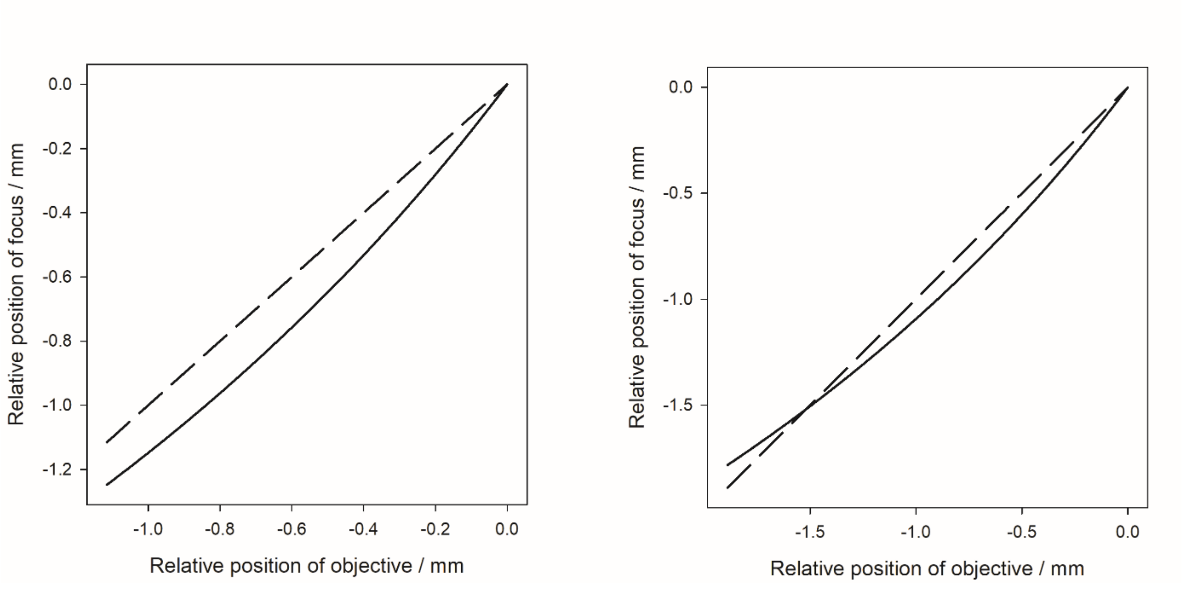

Figure S4. Calculated focus position inside the droplet of the 5 μL DMSO (left) and 10 μL aqueous (right) solutions as a function of the objective position (solid lines). The positions are referenced with respect to the tops of the droplets. Dashed lines correspond to the focus position without the effect of the droplet.

Theoretical dependence of fluorescence intensity on the objective displacement

The measured fluorescence is a superposition of the fluorescence signal from the surface (depicted by a rectangle) and the fluorescence from the solution above the surface. Due to the axial symmetry, the position of any point, from which the fluorescence signal is detected, can be sufficiently characterized by two coordinates, r and z (see Figure S5).

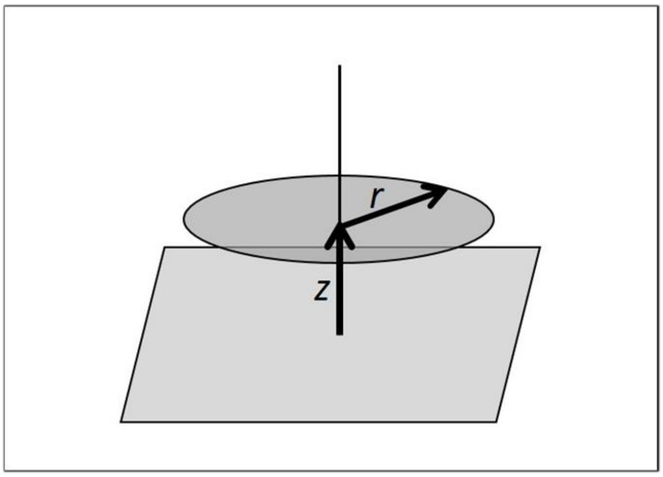

Figure S5. Geometry of the experiment: *r*, *z* positions contributing to the total fluorescence signal.

Let $\varphi(r,z)$ is a contribution of a single molecule in the *r*, *z* position to the total fluorescence signal. This quantity is given by the local spatial density of the exciting radiation, the fluorescence yield and the efficiency of the collecting optical system to get fluorescent radiation from this location to the detector. The total fluorescence signal from the droplet bulk is then

$$I_{vol} = \iint_0^v c' \; N_A \; \varphi(r,z) \; 2 \, \pi \, r \; \mathrm{d}z \; \mathrm{d}r \tag{S8}$$

where v is the droplet height and c' is the actual concentration of the fluorophore inside the droplet.

Supposing the fluorescence from the molecules at the surface is enhanced by a factor ξ, their contribution to the measured fluorescence signal is

$$I_{ads} = \int \sigma \; \xi \; \varphi(r,0) \; 2 \, \pi \, r \; \mathrm{d}r \tag{S9}$$

where σ is the surface density.

The dependence of $\varphi(r,z)$ on *r* (for a particular *z*) is unknown, but we can employ the commonly used Lorentzian function to describe the dependence of the signal on *z*, which means

$$\int \varphi(r,z) \; 2 \, \pi \, r \; \mathrm{d}r = \frac{\varphi_0}{1 + \left(\frac{z}{\delta}\right)^2} \tag{S10}$$

for focus at the surface.

Assuming that the parameter δ does not change when the focus is moved up to *d*, the intensity of the fluorescence signal is

$$\begin{aligned} I(d) = I_{ads}(d) + I_{vol}(d) &= \frac{\sigma \; \xi \; \varphi_0}{1 + \left(\frac{d}{\delta}\right)^2} + c' \; N_A \int_0^v \frac{\varphi_0}{1 + \left(\frac{z-d}{\delta}\right)^2} \, \mathrm{d}z \\ &= \frac{\sigma \; \xi \; \varphi_0}{1 + \left(\frac{d}{\delta}\right)^2} + c' \; N_A \; \delta \; \varphi_0 \left[\tan^{-1}\left(\frac{d}{\delta}\right) + \tan^{-1}\left(\frac{v-d}{\delta}\right)\right] \end{aligned} \tag{S11}$$
